# An Exploratory Analysis of the Effect of Demographic Features on Sleeping Patterns and Academic Stress in Adolescents in China

**DOI:** 10.3390/ijerph19127032

**Published:** 2022-06-08

**Authors:** Alessandro Carollo, Weiyi Chai, Elizabeth Halstead, Dagmara Dimitriou, Gianluca Esposito

**Affiliations:** 1 Department of Psychology and Cognitive Science, University of Trento, 31 Corso Angelo Bettini, 38068 Rovereto, Italy; alessandro.carollo@studenti.unitn.it; 2 Sleep Education and Research Laboratory, UCL Institute of Education, London WC1H 0AA, UK; weiyi.chai@alumni.ucl.ac.uk (W.C.); l.halstead@ucl.ac.uk (E.H.); d.dimitriou@ucl.ac.uk (D.D.)

**Keywords:** adolescents, sleeping patterns, academic stress, demographics, China

## Abstract

Adolescents typically engage in unhealthy lifestyle habits including short sleep and high academic stress. These in turn may have serious impacts on their development. The present study examines the effect of demographic characteristics on sleep patterns and academic stress in adolescents. A sample of 244 (119 females) junior high school adolescents aged between 11 and 16 were recruited from China. The Student Life Stress Questionnaire and the School Sleep Habits Survey were used to assess participants’ sleep habits and academic stress. Multipair and corrected pairwise Kruskal–Wallis tests were conducted to assess the effect of school grade, gender, academic performance level, living situation, single child status, and parental education on adolescents’ sleeping patterns and academic stress. Significant changes in facets of sleeping patterns emerged when examining groups of students in terms of school grade, living situation, and single-child status. Furthermore, caffeine consumption was found to be significantly higher in males, in students with poorer academic performances, and in single-child adolescents. Ultimately, academic stress was modulated by adolescents’ school grade, academic performances, living situation, and single-child status. Developmental trajectories in sleep patterns together with differential exposure to stressors and adopted coping mechanisms are discussed in the manuscript.

## 1. Introduction

Adolescence represents the peak onset age of many mental disorders, such as depression and anxiety [1]. In the United States, for instance, almost 20% of adolescents meet the criteria for a psychiatric diagnosis, and this percentage is expected to increase in the upcoming years [2]. Among other factors, consistent findings have shown that poor sleep habits and higher academic stress not only contribute to endangering adolescents’ successful academic learning but also have an impact on adolescents’ health [3,4]. In the literature, sleep problems (i.e., short sleep duration and low sleep quality) are commonly related to depressive symptoms, anxiety, and externalizing behaviours [5].

### 1.1. Adolescents’ Sleeping Patterns

Sleep patterns reflect a person’s sleep quality (e.g., regenerative and undisturbed sleep), sleep onset latency (i.e., how long the person takes to fall asleep), number of nocturnal awakenings (i.e., how many times the person wakes up at night), and tiredness along with daytime concentration [6]. In both developed and developing countries, adolescents tend to receive shorter sleep durations than recommended [1,7,8]. In fact, while adolescents are recommended to get 9 to 10 h of sleep daily, Hawkins and Takeuchi [8] documented that children in the United States exhibit insufficient sleep duration from the age of 6. Accordingly, recent works report that almost one out of four adolescents from China reports sleep disturbances [9]. Generally, sleep patterns play a significant role in adolescents’ development and in their overall lifestyle [10,11]. Considering the crucial role of sleep on memory consolidation and learning [4,12], poor sleep habits may be detrimental for adolescents’ academic performance [13]. From the literature, it emerges that adolescents’ sleep patterns change according to some well-known objective factors, such as the living environment [14,15,16,17,18], caffeine and stimulants intake [10,19,20,21], and parental socio-economic status [8,17,22]. Moreover, while an increasing number of adolescents seem to enjoy delaying their bedtime [7], engaging in a heavy workload and devoting more time to study play a crucial role in influencing adolescents’ sleep habits. As a matter of fact, heavy workloads tend to induce a delayed bedtime or a reduced sleep duration [23].

### 1.2. Adolescents’ Academic Stress

Academic stress is a well-known common stressor in adolescents’ lives [1,24]. In particular, academic stress stems from the perception of having insufficient resources to face the academic workload or of not being able to reach one’s own or one’s parents’ academic expectations [25]. In particular, the academic burden of adolescents from Asian countries, and especially from China, seems to be heavier as compared to their Western peers. In fact, compared to adolescents from the United States, students from Asian countries tend to spend more time in school and more time doing homework, and they often attend extra classes or private tutoring after school [26]. In Chinese high school students, Liu [27] observed that the heavy academic stress at grade 10 is a predictor of lower students’ intrinsic motivation in the subsequent years. Academic stress is also expected to ultimately affect adolescents’ health [1,28,29,30,31]. Specifically, adolescents reporting high levels of academic stress tend to show a variety of negative outcomes, including poor academic functioning, anxiety, depression, and even suicidal behaviors [1,23,31,32,33,34,35,36]. Thus far, the current literature suggests that adolescents’ academic stress appears to be modulated by many demographic factors, such as age and school grade [37,38], academic environment [18,39], and parental socio-economic status [31,37,40,41].

### 1.3. The Current Study

Although many demographic factors that may influence adolescents’ sleep patterns and academic stress have been identified by recent trends of research, the specific patterns of impact of such demographic features are still debated and underexplored in the literature. Furthermore, most of the literature related to sleep patterns and academic pressure was conducted by examining college students. Considering the differences in most facets of development between young adults and adolescents, what is known about the demographic factors impacting sleep patterns and academic stress cannot be extended to adolescents’ lives yet. Considering the impact of both unhealthy adolescents’ sleep patterns and high levels of academic stress on adolescents’ academic performances, cognitive functioning, and mental health, which poses urgent economical and social challenges to society, it is important to address the aforementioned gaps in the existing literature [13,23,42,43,44,45]. To do so, the current study aims to assess whether and how adolescents’ demographic features are associated with sleep and academic stress profiles. In particular, the study (i) examines which demographic factors (i.e., school grade, gender, academic performance level, living situation, single child status, and parental education) are differentially associated with adolescents’ sleeping patterns and academic stress and (ii) statistically characterizes the specific patterns of association between these demographic factors and participants’ sleep and academic stress profiles. Moreover, given that adolescents in China report heavier academic workloads than their Western counterparts and that academic workload may impact both sleep and stress profiles, the current study focuses on adolescents studying in China.

## 2. Materials and Methods

### 2.1. Study Design

The current study relies on the collection of demographic variables, sleep patterns, and profiles of academic stress in adolescents. All variables were assessed with three self-report questionnaires administered to year 7, 8, and 9 junior high school students in China. Specifically, (i) the Demographic Information Index was used to collect participants’ demographic information, (ii) the Student Life Stress Inventory (SLSI) was adopted to assess participants’ sleep habits and caffeine consumption, and (iii) the School Sleep Habit Survey (SSHS) was chosen to collect academic stress profiles. All instruments were implemented on the first day under the teacher’s supervision.

Before data collection, an ethical review was processed in line with the UCL–Institute of Education’s Research Governance and Ethics Policy. The study was approved by the UCL Research Ethics Committee (approval number Z6364106/201911), and it was conducted in accordance with the Declaration of Helsinki. Prior to participation, schools received an informative invitation letter, and informed consent was obtained from both participants’ parents and participants themselves. All participants were informed that their participation was voluntary, anonymous, and that data confidentiality and privacy would be respected. Participants were allowed to leave aside any questions that they did not want to answer or even withdraw from the study altogether. Eventually, a summary of outcomes was made available to participants who requested it.

### 2.2. Research Instruments

#### 2.2.1. Demographic Information Index

The Demographic Information Index was adopted to collect information about participants’ school grade, gender, academic performance level, living situation, single child status, and parental highest education level.

#### 2.2.2. The School Sleep Habits Survey

The School Sleep Habits Survey [7,46] is a 63-item questionnaire that measures adolescents’ sleep-wake behaviours and typical daytime functioning (Shahid et al., 2011). Specifically, the instrument investigates main sleep-related information, such as weekday and weekend-night bedtimes, wake up times, and total sleep time (TST). Two additional sleep variables are derived to investigate sleep schedule regularity, namely weekend sleep delay (as the difference between weekend bedtime and school-night bedtime) and weekend oversleep (as the difference between weekend total sleep time and school-night total sleep time). The survey also includes a daytime sleepiness scale, which concerns the degree to which the individual struggles to stay awake during the day or to fall asleep in the last two weeks. Furthermore, the survey contains a sleep/wake behaviour problems scale, which refers to erratic sleep/wake problems in the two weeks prior to the assessment. The survey also explores students’ circadian preferences, depressive mood, and caffeine consumption. The School Sleep Habits Survey has not been previously used with a Chinese population. Nevertheless, it has been widely adopted in the literature as an instrument to assess sleep patterns in children and adolescents from non-clinical and clinical populations [5,47,48].

#### 2.2.3. The Student Life Stress Inventory

The Student Life Stress Inventory [28] was adopted to assess the stress derived from students’ academic environment. The questionnaire consists of 51 items divided into two sections: one exploring the types of stressors and the other assessing the reactions to stressors. The two sections are further divided into nine categories. Five categories are included as types of stressors: frustrations, conflicts, pressures, changes, and self-imposed. The remaining four categories belong to the section regarding the different reactions to stressors: physiological, emotional, behavioural, and cognitive. The Student Life Stress Inventory has been widely used in the available literature on academic stress [49,50] and, specifically, it has been previously adopted to assess adolescents’ stress profile in medical students in China (see Liu et al. [51]).

### 2.3. Participants

All participants for this study were recruited from year 7, 8, and 9 of junior high schools in the middle areas of China. A total of 358 (186 males and 172 females; *M age* = 13.37; *SD age* = 1 year) adolescents took part in the study. In the pre-processing phase, to reduce biases and to guarantee the generalizability of the findings, all participants reporting chronic disabilities, medication intake (i.e., Ritalin), and missing data (*n* = 114) were excluded from the analysis. The final sample consisted of 244 students (125 males and 119 females) aged between 11 and 16 years old (*M* = 13.39; *SD* = 1.04 years). Table 1 presents the demographic characteristics of the recruited population.

### 2.4. Data Analysis

The whole data analysis in the current study was conducted with SPSS 26 and Python. All scripts for the data analysis are available at the following link: https://osf.io/xsmhu/ (accessed on 4 June 2022). The influence of demographic factors (i.e., school grade, gender, academic performance level, living situation, single child status, and parental education) on adolescents’ sleeping patterns and academic stress was assessed with a series of multipair Kruskal–Wallis tests [52,53,54,55]. In particular, in each multipair Kruskal–Wallis test, one demographic variable was considered as the factor, and one variable regarding sleeping patterns or academic stress was considered as the dependent variable. Variables regarding participants’ sleeping patterns were weekday bedtimes, wake up times, and total sleep time; weekend bedtimes, wake up times, and total sleep time; weekend sleep delay, weekend oversleep, daytime sleepiness, sleep/wake behaviour problems, circadian preference, and caffeine consumption. Two Bonferroni corrections were computed to adjust the significance levels and to control for false-positive results (i.e., type I error) in a conservative way [56]. The first Bonferroni correction was computed to interpret the results regarding the influence of demographic variables on sleeping patterns. Here, significance levels were corrected at *p* < 0.004. The second Bonferroni correction was computed for the analysis of the effect of demographic variables on academic stress. In this case, significance levels were corrected at *p* < 0.005. When significant results emerged from the multipair comparisons, post-hoc Kruskal–Wallis tests were computed to compare the groups in a pairwise fashion (as in Carollo et al. [57]). After the detection of significant results, the eta-squared was computed to assess the magnitude of the effects [58].

## 3. Results

Descriptive statistics for participants’ sleep profiles and academic stress are reported in Table S1 of the Supplementary Materials.

### 3.1. The Influence of Gender on Sleep Patterns and Academic Stress

A series of Kruskal–Wallis tests was computed to test the effect of gender on each SSHS dimension of interest. Results showed that males tended to consume significantly more caffeine than females (H[1, 244] = 20.090, *p* = 0.000007, $\eta^{2}$ = 0.079).

Conversely, no significant difference among males and females emerged in terms of their academic stress.

### 3.2. The Influence of School Grade on Sleep Patterns and Academic Stress

**Figure 1:**
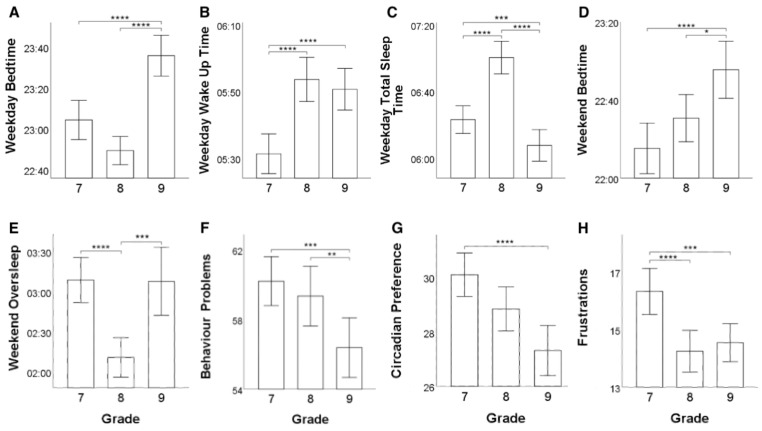
Bar plots representing the significant differences in terms of sleep patterns and academic stress across school grade groups. Specifically, the figure reports differences across school grades in terms of: (**A**) weekday bedtime; (**B**) weekday wake up time; (**C**) weekday total sleep time; (**D**) weekend bedtime; (**E**) weekend oversleep; (**F**) sleep/wake behaviour problems; (**G**) circadian preference; and (**H**) stress derived from frustrations (* *p* < 0.017; ** *p*< 0.01; *** *p*< 0.001; **** *p*< 0.0001).

As regards adolescents’ sleeping patterns, significant differences emerged from the Kruskal–Wallis tests in terms of weekday bedtimes (H[2, 244] = 62.099, *p* = 3.28 × 10${}^{-14}$, $\eta^{2}$ = 0.249), weekday wake up times (H[2, 244] = 44.065, *p* = 2.70 × 10${}^{-10}$, $\eta^{2}$ = 0.175), weekday total sleep time (H[2, 244] = 57.453, *p* = 3.34 × 10${}^{-13}$, $\eta^{2}$ = 0.230), weekend bedtimes (H[2, 244] = 17.692, *p* = 0.000144, $\eta^{2}$ = 0.065), weekend oversleep (H[2, 244] = 24.017, *p* = 0.000006, $\eta^{2}$ = 0.091), sleep/wake behavioural problems (H[2, 244] = 12.189, *p* = 0.002, $\eta^{2}$ = 0.042), and circadian preference (H[2, 244] = 18.059, *p* = 0.00012, $\eta^{2}$ = 0.067). Post-hoc pairwise Kruskal–Wallis tests were conducted with p value adjusted with Bonferroni correction at *p* < 0.017. On the one hand, it emerged that adolescents in grade 9 showed later weekday bedtimes than adolescents in both grade 7 (H[1, 244] = 33.992, *p* = 5.53 × 10${}^{-9}$, $\eta^{2}$ = 0.145) and grade 8 (H[1, 244] = 56.780, *p* = 4.87 × 10${}^{-14}$, $\eta^{2}$ = 0.239; see Figure 1A). On the other hand, grade 7 students prefer to wake up earlier than adolescents in grade 8 (H[1, 244] = 31.267, *p* = 2.25 × 10${}^{-8}$, $\eta^{2}$ = 0.133) and grade 9 (H[1, 244] = 35.121, *p* = 3.10 × 10${}^{-9}$, $\eta^{2}$ = 0.149; see Figure 1B). Thus, overall, students in grade 8 tend to sleep more than students in grade 7 (H[1, 244] = 27.674, *p* = 1.43 × 10${}^{-7}$, $\eta^{2}$ = 0.118) and grade 9 (H[1, 244] = 44.684, *p* = 2.32 × 10${}^{-11}$, $\eta^{2}$ = 0.189). Moreover, students in grade 9 tend to sleep less than students in grade 7 (H[1, 244] = 7.542, *p* = 0.006, $\eta^{2}$ = 0.035; see Figure 1C). Likewise, students in grade 9 tend to prefer later weekend bedtimes than both grade 7 (H[1, 244] = 17.030, *p* = 3.67 × 10${}^{-5}$, $\eta^{2}$ = 0.075) and grade 8 students (H[1, 244] = 6.434, *p* = 0.011, $\eta^{2}$ = 0.031; see Figure 1D). Overall, students in grade 8 oversleep less than grade 7 (H[1, 244] = 22.750, *p* = 1.84 × 10${}^{-6}$, $\eta^{2}$ = 0.098) and grade 9 students (H[1, 244] = 13.015, *p* = 0.0003, $\eta^{2}$ = 0.058) during weekends (see Figure 1E). Furthermore, students in grade 9 showed fewer sleep/wake behavioural problems than students in grade 8 (H[1, 244] = 8.002, *p* = 0.005, $\eta^{2}$ = 0.037) and grade 7 (H[1, 244] = 10.377, *p* = 0.001, $\eta^{2}$ = 0.047; see Figure 1F) and a higher preference for staying up later than grade 7 (H[1, 244] = 17.892, *p* = 2.33 × 10${}^{-5}$, $\eta^{2}$ = 0.078; see Figure 1G).

Adolescents’ academic stress profiles resulted to be significantly different among grade groups in terms of stress derived from frustrations (H[2, 244] = 17.814, *p* = 0.000135, $\eta^{2}$ = 0.066). Post-hoc pairwise Kruskal–Wallis tests with a significance threshold set at *p* < 0.017 showed that grade 7 students report more stress derived from frustration than grade 8 (H[1, 244] = 14.781, *p* = 0.0001, $\eta^{2}$ = 0.065) and grade 9 (H[1, 244] = 11.229, *p* = 0.0008, $\eta^{2}$ = 0.051) students (see Figure 1H).

### 3.3. The Influence of Being a Single-Child on Sleep Patterns and Academic Stress

Adolescents with no siblings reported significantly later weekday wake up times (H[1, 244] = 11.985, *p* = 0.001, $\eta^{2}$ = 0.045) and less stress derived from frustrations (H[1, 244] = 8.706, *p* = 0.003, $\eta^{2}$ = 0.032) as compared to adolescents with siblings.

### 3.4. The Influence of the Living Situation on Sleep Patterns and Academic Stress

**Figure 2:**
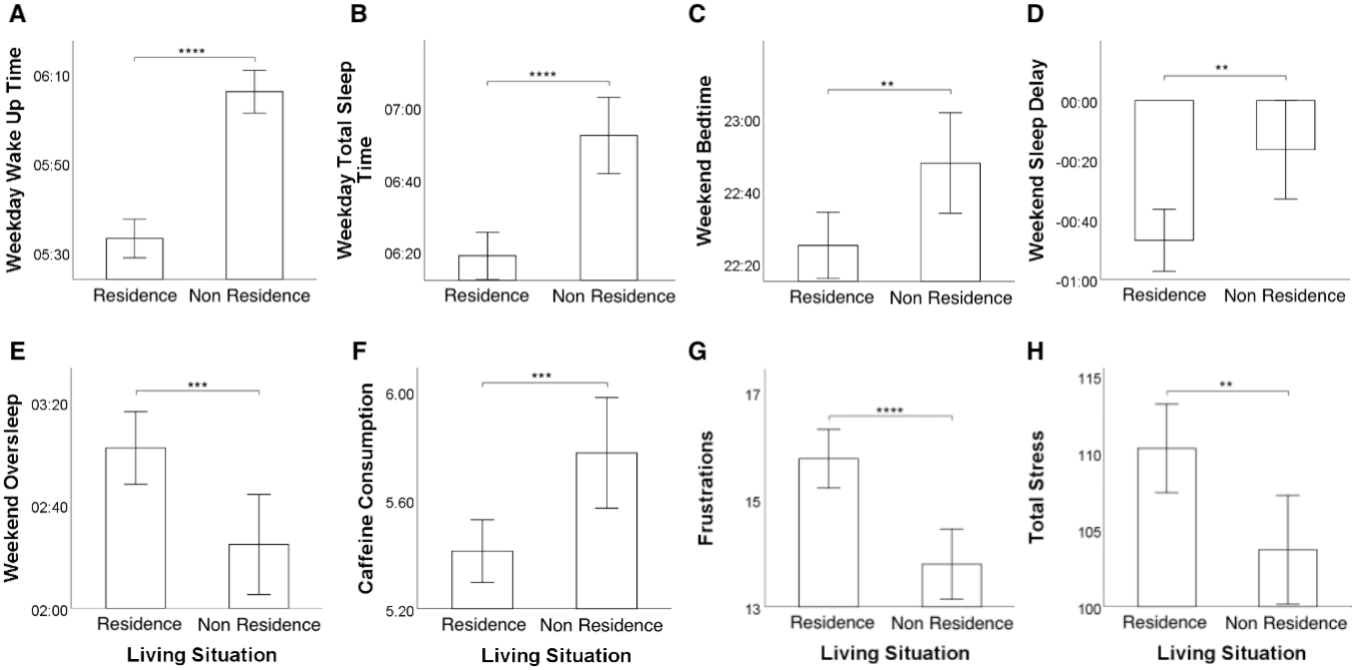
Bar plots representing the significant differences in terms of sleep patterns and academic stress across living situation groups. Specifically, the figure reports differences across living situation groups in terms of: (**A**) weekday wake up time; (**B**) weekday total sleep time; (**C**) weekend bedtime; (**D**) weekend sleep delay; (**E**) weekend oversleep; (**F**) caffeine consumption; (**G**) stress derived from frustrations; and (**H**) total stress (** *p* < 0.01; *** *p*< 0.001; **** *p*< 0.0001).

Kruskal–Wallis tests on sleeping pattern variables showed that adolescents living in dormitories tend to report earlier weekday wake up times (H[1, 244] = 92.333, *p* = 7.32 × 10${}^{-22}$, $\eta^{2}$ = 0.377; see Figure 2A), less weekday total sleep time (H[1, 244] = 26.871, *p* = 2.17 × 10${}^{-7}$, $\eta^{2}$ = 0.107; see Figure 2B), earlier weekend bedtimes (H[1, 244] = 8.703, *p* = 0.003, $\eta^{2}$ = 0.032; see Figure 2C), less weekend sleep delay (H[1, 244] = 9.994, *p* = 0.002, $\eta^{2}$ = 0.037; see Figure 2D), more weekend oversleep (H[1, 244] = 11.605, *p* = 0.001, $\eta^{2}$ = 0.044; see Figure 2E), and lower caffeine consumption (H[1, 244] = 10.187, *p* = 0.001, $\eta^{2}$ = 0.038; see Figure 2F) than their counterparts not living in dormitories.

In terms of academic stress, students living in residences showed higher stress derived from frustrations (H[1, 244] = 19.099, *p* = 0.000012, $\eta^{2}$ = 0.074; see Figure 2G) and total stress (H[1, 244] = 9.689, *p* = 0.002, $\eta^{2}$ = 0.036; see Figure 2H).

### 3.5. The Influence of Academic Performance Levels on Sleep Patterns and Academic Stress

Adolescents’ academic performance was found to be significantly associated with differential levels of caffeine intake (H[3, 244] = 19.294, *p* = 0.000238, $\eta^{2}$ = 0.068). Post-hoc tests with significance levels adjusted at *p* < 0.008 showed that adolescents with D grades tended to consume more caffeine than adolescents with A grades (H[1, 244] = 17.700, *p* = 2.58 × 10${}^{-5}$, $\eta^{2}$ = 0.077).

In addition, academic stress profiles significantly differed with regards to adolescents’ academic performances in terms of cognitive reactions to stress (H[3, 244] = 14.469, *p* = 0.002, $\eta^{2}$ = 0.048). Specifically, post-hoc tests showed that adolescents with D performances tended to have higher cognitive reactions to stress than adolescents with A performances (H[1, 244] = 11.917, *p* = 0.0006, $\eta^{2}$ = 0.053) and adolescents with C performances (H[1, 244] = 7.263, *p* = 0.007, $\eta^{2}$ = 0.034).

### 3.6. The Influence of Parental Education on Sleep Patterns and Academic Stress

The impact of parental education on sleeping patterns and academic stress was controlled, with no documented significant results.

Table 2 and Table 3 summarize the results from the computed multipair Kruskal–Wallis tests.

## 4. Discussion

The current study investigated the relationship between adolescents’ demographic information, sleep patterns, and academic stress. First, the study identified specific demographic information that is differentially associated with sleep patterns and academic stress in adolescents in China. Second, it statistically outlined the specific patterns of association of such demographic factors with adolescents’ sleep and academic stress profiles. Specifically, it emerged that adolescents’ school grade, living situation, and single-child status are associated with their sleeping patterns. Furthermore, male gender, poorer academic performance level, and living situation (i.e., students not living in dormitories) are all associated with higher amounts of caffeine intake, posing an increased risk for unhealthy sleep behaviours [59]. Finally, school grade, academic performance, living situation, and single-child status are related to the adolescents’ academic stress.

Although most of these demographic influences are well-known in the available sleep literature and academic stress [60,61,62,63,64,65,66,67], less explored is the specific way in which demographic variables influence and are associated with adolescents’ lives. In the current paper, it emerged that, as adolescents progress in their school grade, on weekdays, they tend to prefer later bedtimes and wake up times. Likewise, at school grade 9, students tend to prefer later bedtime also on weekends. A transaction to later bedtimes is found across adolescents from all over the world, both from Western or Eastern countries [64,68,69]. As discussed by Gariepy et al. [66], such transactions on sleeping patterns seem to be a consequence of biological, behavioural, and social modifications in adolescents’ lives [70,71,72,73]. Furthermore, in the current study, it emerged that students in grade 8 report significantly higher total sleep time during the weekdays as they are likely to be in the middle of their transaction phase of sleeping habits. As a consequence, the same adolescents also report a lower amount of weekend oversleep, testifying once again the close link between total sleep during the week and the degree of sleep overcompensation during the weekend [74,75,76]. When they reach school grade 9, adolescents tend to report fewer sleep/wake behaviour problems and higher preferences for an eveningness schedule than before. The shift from morningness to eveningness during adolescents’ puberty finds agreement in the available literature [77].

Moreover, living in a residence was associated with reduced adolescents’ weekday wake up times and lower total sleep duration during the week, likely because of the noise of the dormitories [15]. Once again, insufficient amounts of sleep during the weekdays are overcompensated during the weekend by adolescents. In fact, adolescents living in dormitories report earlier weekend bedtimes, less weekend sleep delay, and more weekend oversleeping than their counterparts not living in dormitories. As regards their sleeping patterns, single-child adolescents tend to have later wake up times during the weekdays than adolescents with siblings.

When examining the ways in which demographic factors are specifically associated with academic stress, we found that frustration-like stressors are more common among grade 7 students, students living in dormitories, and non-single-child adolescents. In fact, more frustrations can arise when facing many new challenges, for which the individual may have not developed the adequate coping strategies yet, as might be the case for younger adolescents [78]. Moreover, frustrations may arise in the process of adaptation to life in a students’ dormitory [79] or even from the relationships within the household [80]. Not only did students living in dormitories report higher stressors in terms of frustrations, but also they tended to show higher levels of total stress than their counterparts. Finally, poorer academic performances are associated with higher cognitive reactions to stressors. It is likely that cognitive reactions may arise as an efficient strategy to cope with the stress of having low academic performances and preserving one’s self-esteem [81].

### Study Limitations and Future Research Perspectives

In conclusion, the current study shed light on specific demographic variables that have an impact on adolescents’ sleep patterns and academic stress. Despite some initial trends on the influence of demographic factors on adolescents’ lives having emerged, some limitations need to be taken into account. For instance, in regards to the analytical approach, a large number of participants (*n* = 144) was excluded prior to analysis due to reports of chronic disabilities, medication intake, and missing data. When doing so, no sensitivity analysis was conducted to assess the impact of the data removal on the obtained results. Moreover, before conducting the Kruskal–Wallis tests, the distribution and variability of observations across groups were not statistically characterized. The study design carries some intrinsic limitations as well. In fact, all variables were investigated through self-report questionnaires and in a cross-sectional fashion. To do so, all data obtained from self-report questionnaires were treated as manifest variables, although being intrinsically latent. Furthermore, the cross-sectional nature of the study does not allow us to interpret the role played by demographic features in adolescents’ life in a causal fashion. Although no causal interpretation can be obtained, the approach adopted in the study allowed us to uncover some initial trends on the way in which demographic factors influence adolescents’ lives. Future lines of research could extend the results by using different methods to assess sleep and stress profiles and to address the limitations of the current study. For instance, the influence of demographic variables on sleep patterns and stress could be documented longitudinally and/or at the physiological level with instruments such as actigraphy or neuro-imaging techniques. The integration of self-report and physiological techniques would allow us to increase the validity and reliability of the results of the current paper and would lead to a more complete picture of the way in which sleep and stress profiles change during adolescence based on individuals’ demographic characteristics. Not only would such a line of research lead to a more profound understanding of the variations in adolescents’ sleep and stress profiles, it would also have translational implications since new insights would optimize the design of more effective and customized sleep improvement and stress management interventions.

## Figures and Tables

**Table 1:** Prevalence of main demographic variables in the sample.

	N	%
**Gender**		
Male	125	51.20
Female	119	48.80
**School Grade**		
7	83	34.00
8	84	34.40
9	77	31.60
**Single Child Status**		
Yes	43	17.60
No	201	82.40
**Living Situation**		
Residence	155	63.50
Non Residence	99	36.50
**Academic Performance Level**		
A	101	41.40
B	57	23.40
C	41	16.80
D	45	18.40
**Parental Education**		
Secondary School	54	22.10
High School	122	50.00
College	68	27.90

**Table 2:** Results of the multipair Kruskal–Wallis tests with sleep dimensions as dependent variables and demographic information as single factors. Sleep dimensions of interest were weekday bedtime (WD BT), weekday wake up time (WD WUT), weekday total sleep time (WD TST), weekend bedtime (WE BT), weekend wake up Time (WE WUT), weekend total sleep time (WE TST), weekend sleep delay (WE SD), weekend oversleep (WE OS), daytime sleepiness (DTS), sleep/wake behaviour problems (S/W BP), circadian preference (CP), and caffeine consumption (CC). (* *p* < 0.004; ** *p*< 0.001; *** *p*< 0.0001).

	WD BT	WD WUT	WD TST	WE BT	WE WUT	WE TST	WE SD	WE OS	DTS	S/W BP	CP	CC
**Gender**	0.79	0.88	0.72	5.33	2.00	5.26	4.20	3.61	0.91	1.07	1.02	20.09 ***
**School Grade**	62.10 ***	44.07 ***	57.45 ***	17.69 ***	5.91	4.48	8.95	24.02 ***	5.42	12.19 *	18.06 **	2.99
**Single Child Status**	2.94	11.99 **	3.48	7.38	0.03	3.18	3.25	5.09	6.27	0.46	1.44	1.28
**Living Situation**	0.68	92.33 ***	26.87 ***	8.70 *	1.85	0.33	9.99 *	11.61 **	2.12	0.19	2.36	10.19 **
**Academic Performance Level**	7.43	3.10	4.72	4.06	9.83	3.39	4.17	3.43	6.19	1.82	2.32	19.29 **

**Table 3:** Results of the multipair Kruskal–Wallis tests with academic stress dimensions as dependent variables and demographic information as single factors. Stress dimensions of interest were frustrations (Frust), conflicts, pressure, changes, self-imposed, physiological (Physio), emotional, behavioural (Behav), cognitive, and total stress (TS). (* *p* < 0.005; ** *p*< 0.001; *** *p*< 0.0001).

	Frust	Conflicts	Pressure	Changes	Self-Imposed	Physio	Emotional	Behav	Cognitive	TS
**School Grade**	17.81 **	5.37	1.58	0.19	6.03	3.34	6.81	4.44	0.48	10.10
**Single Child Status**	8.71 *	4.77	0.08	0.02	1.80	0.06	3.28	0.03	0.02	2.59
**Living Situation**	19.10 ***	0.07	3.35	0.37	2.70	0.35	3.77	5.01	0.53	9.69 *
**Academic Performance Level**	3.63	2.07	5.58	7.78	5.91	5.82	4.25	5.37	14.47 *	4.34

## Data Availability

The data presented in this study are available on request from Professor Dagmara Dimitriou. Scripts for the data analysis are available at the following link: https://osf.io/xsmhu/ (accessed on 4 June 2022).

## Supplementary Materials

The following supporting information can be downloaded at https://www.mdpi.com/article/10.3390/ijerph19127032/s1. Table S1: Descriptive statistics for sleep patterns and academic stress variables.

## Abbreviations

The following abbreviations are used in this manuscript:

SSHS	School Sleep Habits Survey
SLSI	Student Life Stress Inventory
TST	Total Sleep Time
