# Supplementary material for: An Exploratory Analysis of the Effect of Demographic Features on Sleeping Patterns and Academic Stress in Adolescents in China
Source: Int J Environ Res Public Health. 2022 Jun 8;19(12):7032. doi: 10.3390/ijerph19127032 (PMC9222687; doi:10.3390/ijerph19127032)
Supplement: Supplementary file 1 [file ijerph-19-07032-s001.zip › ijerph-1729079-supplementary.pdf]

1. Supplementary Tables

1.1. Table S1: Descriptive statistics for sleep patterns and academic stress variables.

Table S1. Descriptive statistics in terms of Minimum (Min), Maximum (Max), Mean, and Standard Deviation (SD) for sleep patterns and academic stress in N = 244 adolescents.

Variable	Min	Max	Mean	SD
**Sleep Patterns**				
Weekdays Bedtime	21:00	02:00	23:09	0:44
Weekdays Wake Up Time	05:00	08:00	05:45	0:29
Weekdays Total Sleep Time	4h 30	10h 31	6h 30	0h 46
Weekend Bedtime	19:00	01:30	22:33	01:01
Weekend Wake Up Time	05:00	14:00	08:07	01:15
Weekend Total Sleep Time	05h 00	14h 00	09h 19	01h 21
Weekend Sleep Delay	-4h 50	4h 30	- 0h 35	1h 11
Weekend Oversleep	-1h 00	9h 00	2h 48	1h 30
Daytime Sleepiness	10.00	32.00	16.57	4.15
Sleep/Wake Behaviour Problems	24.00	75.00	58.70	7.43
Circadian Preference	16.00	37.00	28.78	3.92
Caffeine consumption	5.00	9.00	5.55	0.83
**Academic Stress**				
Frustrations	7.00	25.00	15.05	3.42
Conflicts	3.00	15.00	6.30	2.42
Pressures	4.00	18.00	10.75	2.69
Changes	3.00	41.00	7.57	3.06
Self-imposed	8.00	28.00	17.95	3.97
Physiological	14.00	45.00	22.60	5.92
Emotional	5.00	23.00	11.59	3.70
Behavioural	8.00	33.00	11.79	3.58
Cognitive	2.00	10.00	6.03	2.02
Total stress	65.00	164.00	107.91	17.78
